# Photobiomodulation Reduces the Cytokine Storm Syndrome Associated with COVID-19 in the Zebrafish Model

**DOI:** 10.3390/ijms24076104

**Published:** 2023-03-24

**Authors:** Ivana F. Rosa, Ana P. B. Peçanha, Tábata R. B. Carvalho, Leonardo S. Alexandre, Vinícius G. Ferreira, Lucas B. Doretto, Beatriz M. Souza, Rafael T. Nakajima, Patrick da Silva, Ana P. Barbosa, Leticia Gomes-de-Pontes, Camila G. Bomfim, Glaucia M. Machado-Santelli, Antonio Condino-Neto, Cristiane R. Guzzo, Jean P. S. Peron, Magaiver Andrade-Silva, Niels O. S. Câmara, Anali M. B. Garnique, Renata J. Medeiros, Fausto K. Ferraris, Leonardo J. G. Barcellos, Jose D. Correia-Junior, Jorge Galindo-Villegas, Mônica F. R. Machado, Angela Castoldi, Susana L. Oliveira, Camila C. Costa, Marco A. A. Belo, Giovane Galdino, Germán G. Sgro, Natalia F. Bueno, Silas F. Eto, Flávio P. Veras, Bianca H. V. Fernandes, Paulo R. S. Sanches, Eduardo M. Cilli, Guilherme Malafaia, Rafael H. Nóbrega, Aguinaldo S. Garcez, Emanuel Carrilho, Ives Charlie-Silva

**Affiliations:** 1 Department of Structural and Functional Biology, Institute of Biosciences, São Paulo State University (UNESP), Botucatu 01049-010, Brazil; 2 Department of Orthodontics, São Leopoldo Mandic College, Campinas 13045-755, Brazil; 3 Instituto de Química de São Carlos, Universidade de São Paulo, São Carlos 13566-590, Brazil; 4 The National Institute of Science and Technology in Bioanalyses, INCTBio, Campinas 13083-970, Brazil; 5 Institute of Biomedical Sciences, University of São Paulo (USP), São Paulo 05508-220, Brazil; 6 Oswaldo Cruz Foundation (Fiocruz), Rio de Janeiro 21040-900, Brazil; 7 Laboratório de Fisiologia de Peixes, Programa de Pós-Graduação em Bioexperimentação, Escola de Ciências Agrárias, Inovação e Negócios, Universidade de Passo Fundo, Passo Fundo 99052-900, Brazil; 8 Institute of Biomedical Sciences, Federal University Minas Gerais, Belo Horizonte 31270-901, Brazil; 9 Department of Genomics, Faculty of Biosciences and Aquaculture, Nord University, 8026 Bodø, Norway; 10 Biological Sciences Special Academic Unit, Federal University of Jatai, Jatai 75804-020, Brazil; 11 Keizo Asami Institute, Federal University of Pernambuco, Recife 50670-901, Brazil; 12 School of Agricultural and Veterinary Sciences, São Paulo State University (UNESP), Jaboticabal 14884-900, Brazil; 13 Institute of Motricity Sciences, Department of Physical Therapy, Federal University of Alfenas, Alfenas 37133-840, Brazil; 14 Departamento de Ciências Biomoleculares, Faculdade de Ciências Farmacêuticas de Ribeirão Preto, Universidade de São Paulo, São Paulo 14040-900, Brazil; 15 Integrated Structural Biology Platform, Carlos Chagas Institute, FIOCRUZ Paraná, Curitiba 81310-020, Brazil; 16 Center of Innovation and Development, Laboratory of Development and Innovation Butantan Institute, São Paulo 69310-000, Brazil; 17 Faculty of Medicine, University of São Paulo (USP), Ribeirão Preto 14040-900, Brazil; 18 Laboratory of Genetic and Sanitary Control, Technical Board of Support for Teaching and Research, Faculty of Medicine, University of Sao Paulo, São Paulo 01246-903, Brazil; 19 Department of Biochemistry and Organic Chemistry, Institute of Chemistry, São Paulo State University (UNESP), Araraquara 14800-060, Brazil; 20 Laboratory of Toxicology Applied to the Environment, Goiano Federal Institute, Urutaí Campus, Urutaí 75790-000, Brazil

**Keywords:** cytokine storms, oxidative stress, COVID-19, rSpike, photobiomodulation, zebrafish

## Abstract

Although the exact mechanism of the pathogenesis of coronavirus SARS-CoV-2 (COVID-19) is not fully understood, oxidative stress and the release of pro-inflammatory cytokines have been highlighted as playing a vital role in the pathogenesis of the disease. In this sense, alternative treatments are needed to reduce the level of inflammation caused by COVID-19. Therefore, this study aimed to investigate the potential effect of red photobiomodulation (PBM) as an attractive therapy to downregulate the cytokine storm caused by COVID-19 in a zebrafish model. RT-qPCR analyses and protein–protein interaction prediction among SARS-CoV-2 and *Danio rerio* proteins showed that recombinant Spike protein (rSpike) was responsible for generating systemic inflammatory processes with significantly increased levels of pro-inflammatory (*il1b*, *il6*, *tnfa*, and *nfkbiab*), oxidative stress (*romo1*) and energy metabolism (*slc2a1a and coa1*) mRNA markers, with a pattern similar to those observed in COVID-19 cases in humans. On the other hand, PBM treatment was able to decrease the mRNA levels of these pro-inflammatory and oxidative stress markers compared with rSpike in various tissues, promoting an anti-inflammatory response. Conversely, PBM promotes cellular and tissue repair of injured tissues and significantly increases the survival rate of rSpike-inoculated individuals. Additionally, metabolomics analysis showed that the most-impacted metabolic pathways between PBM and the rSpike treated groups were related to steroid metabolism, immune system, and lipid metabolism. Together, our findings suggest that the inflammatory process is an incisive feature of COVID-19 and red PBM can be used as a novel therapeutic agent for COVID-19 by regulating the inflammatory response. Nevertheless, the need for more clinical trials remains, and there is a significant gap to overcome before clinical trials can commence.

## 1. Introduction

The SARS-CoV-2 virus emerged and rapidly spread worldwide since it was first reported at the end of 2019; now it is still a global health threat due to the emergence of new variants of concern (VOC) [1,2,3]. The SARS-CoV-2 virus infects human target cells via its transmembrane Spike glycoprotein, which is required for virus recognition and attachment to host cells [4,5]. Additionally, the angiotensin-2 converting enzyme (ACE2), which is present in various organs, such as the kidneys, heart, intestines, liver, lungs, muscles, and testicles, has recently been described as a critical host-cell-membrane receptor essential for the transmission of SARS-CoV-2 [6,7,8,9].

Although the exact mechanism of the pathogenesis of COVID-19 is not fully understood, oxidative stress and the release of pro-inflammatory cytokines, or “cytokine storms”, have been highlighted as playing a vital role in the pathogenesis of the disease [10,11,12]. Essentially, the oxidative stress induced by the virus is mediated by an immune response, which stimulates the secretion of critical pro-inflammatory factors that promote acute inflammation in several organs [11,13]. According to recent studies, the secretion of cytokines, such as interleukin (IL)-1β, IL6, and IL-10, and mediators such as TNF-α have been significantly stimulated in patients with COVID-19 and are associated with rapid disease progression in high-risk patients [14,15,16,17,18,19].

Since inflammatory cytokines play an essential role in the pathogenesis of COVID-19, and we have an inability to predict which individuals are prone to the “cytokine storm” and disease aggravation, alternative treatments are needed to reduce the level of inflammation and pathology caused by COVID-19 [12,20]. Therefore, photobiomodulation (PBM) with a low-level laser is an alternative treatment for tissue pathologies [21].

PBM has been used extensively in medicine to treat pathological tissues characterized by some degree of alteration, such as scarring or inflammation [22,23,24]. The use of the laser shows several biomodulatory effects, including increased tissue repair, local circulation, and collagen synthesis [25,26,27]. Additional benefits of PBM use include the modulation of pro-inflammatory cytokines, such as TNF-α, *IL1β*, and IL6 [21,28,29,30].

Hence, PBM may be an attractive complementary therapy to combat the pathology and inflammation processes caused by COVID-19. In this study, we demonstrated the induction of inflammatory responses by the recombinant Spike protein (rSpike), while PBM significantly increased cellular and tissue repair in various zebrafish tissues. Strikingly, the phototherapy resulted in a decrease in mRNA expression of some pro-inflammatory markers (*il1b*, *il6*, *tnfa*, and *nfkbiab*) and oxidative stress modulators (*romo1*, *coa1*, and *slc2a1a*) in the tissues affected by the cytokine storm syndrome (CSS) associated with COVID-19.

## 2. Results

### 2.1. rSpike Is Enough to Trigger the COVID-19-Associated Cytokine Storm Syndrome in Zebrafish

**Figure 1:**
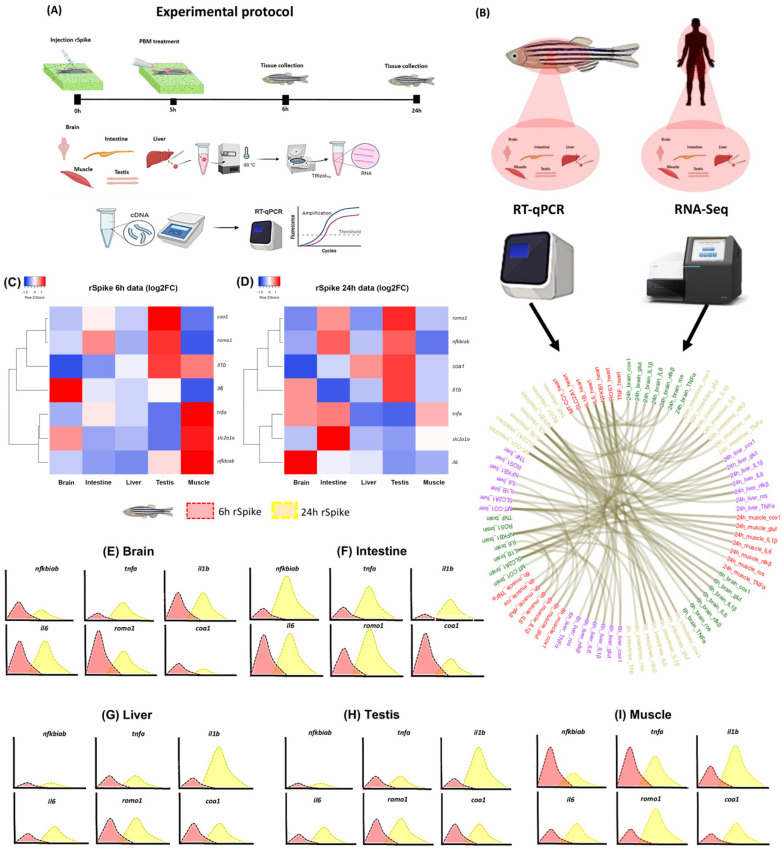
Overview of the transcriptional signature of COVID-19-associated cytokine storm syndrome in a zebrafish model. (**A**) Experimental protocol; (**B**) Chord diagram of the transcriptional signature and evolution of fold-change in the rSpike group in different tissue with the comparison of RNA-seq expression analysis in human tissue with COVID-19 (*n* = 30) versus without COVID-19 (*n* = 36) (from data set); (**C**) Heatmap illustrating the fold-change of relative expression levels of related genes in the brain, intestine, liver, testis, and muscle treated with rSpike at 6 h; (**D**) Heatmap illustrating the fold-change of relative expression levels of related genes in the brain, intestine, liver, testis, and muscle treated with rSpike at 24 h; Curve chart illustrating the relative expression levels of genes in (**E**) the brain, (**F**) intestine, (**G**) liver, (**H**) testis, and (**I**) muscle. The *x*-axis represents the different times (red 6 h and yellow 24 h), and the *y*-axis represents each gene’s relative expression.

To confirm our hypothesis that rSpike triggers inflammatory processes in zebrafish, we analyzed the expression pattern of interleukin and factors involved in cellular processes in different tissues treated with rSpike (Figure 1A,C–I) and analysed RNA-seq expression in human tissue with COVID-19 (Figure 1B). The protein–protein interaction prediction among SARS-CoV-2 and *D. rerio* proteins according to subcellular location (cytoplasm, membrane, and nucleus) predicted interactions with 778 proteins for the cytoplasm, 2917 proteins for the membrane, and 1141 proteins for the nucleus (Table S1). In the GO analysis we identified 48 related terms for the cytoplasm, 5 representative terms for the membrane, and 22 representative terms for the nucleus (Table S2). These data emphasized the biological pathways that can be modifying the normal function in an infection with SARS-CoV-2 and the alteration of the expression of the related genes. In general, the biological pathways emphasized the defense system, signal transduction, cellular programming, and cellular response (Figure S1; Table S1). Additionally, functional enrichment for the nucleus subcellular location retrieved the transcription factor Pax-5 (TF:M00144, padj: 6.29 × 10^−4^).

The results of the chord diagram in human tissue with COVID-19 showed the transcriptional signature of pro-inflammatory markers, such as *nfkbiab*, *tnfa*, *il1b*, and *il6*, including the genes *romo1* (reactive oxygen species), *coa1* (cytochrome c oxidase 1), and *slc2a1a* (glucose transporter 2) involved in the processes of reactive oxygen species production, mitochondrial respiratory chain, and cell membrane glucose transport (Figure 1B). Similarly in zebrafish, treatment with rSpike significantly increased the mRNA levels of *nfkbiab*, *il1b*, and *il6* after 6 and 24 h in all tissue (Figure 1C–I). Additionally, *romo1*, *coa1* and *slc2a1a* were also positively regulated by rSpike after 6 and 24 h (Figure 1C–I). Similar to interleukins, after 6 and 24 h rSpike promoted a significant increase in tumor necrosis factor (tnfa) in the brain, intestines, and muscle (Figure 1C–F,I).

### 2.2. PBM Downregulates Inflammatory Response in COVID-19 Zebrafish Model

**Figure 2:**
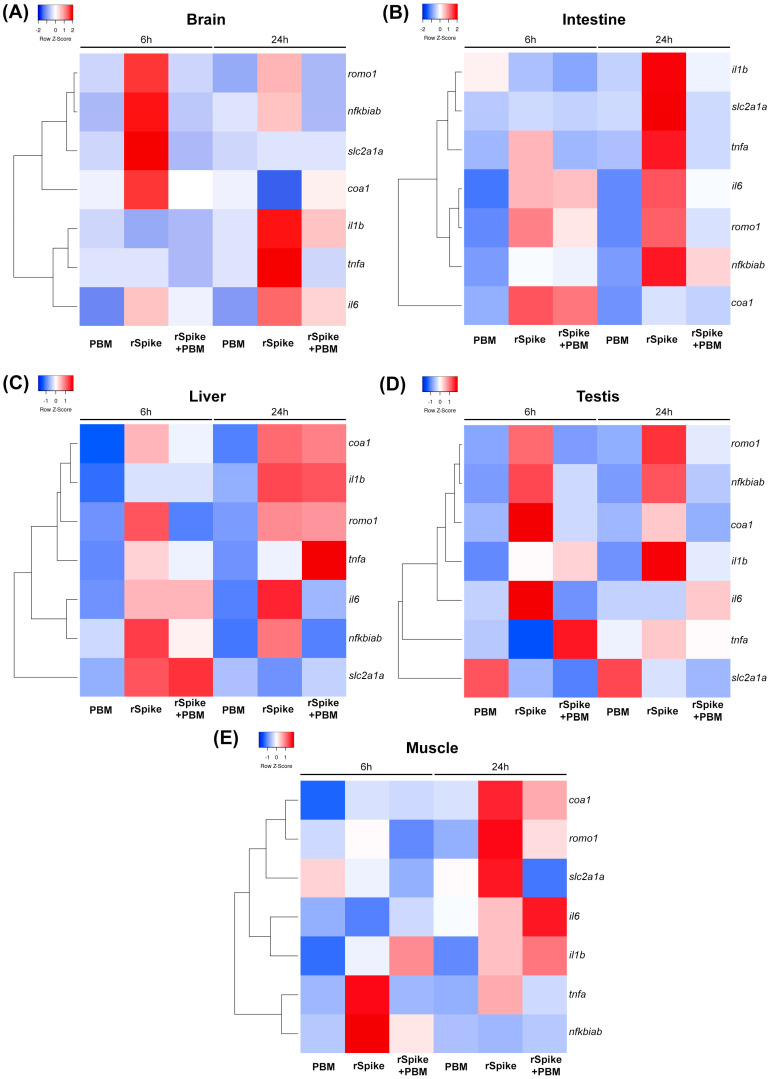
PBM with low-level laser as a potential therapeutic in pathologies originating from SARS-COV-2. Different tissue in (**A**) the brain, (**B**) intestine, (**C**) liver, (**D**) testis, and (**E**) muscle of zebrafish males, subjected to low-level laser (6 h and 24 h); injected with recombinant Spike protein (rSpike) and subsequently subjected to PBM (6 h and 24 h). Each colored cell on the map represents the normalized relative gene expression value for each treatment and sample. In each heat map, genes (rows) are grouped hierarchically using Pearson’s correlation as the distance metric.

The pattern of PBM treatment was also observed in the corresponding heatmaps for the gene expression in all tissues (Figure 2A–E) and the statistical analysis between the groups was demonstrated in Figures S2–S6. The qPCR analysis demonstrated that PBM via low-level laser treatment in individuals subjected to the rSpike significantly decreased the expression of *romo1*, *nfkbiab*, *il1b*, *il6*, and *slc2a1a* in the brain tissue after 6 h compared to the rSpike group only (Figure 2A and Figure S2). Only *romo1*, *nfkbiab*, and *tnfa* showed a significant decrease in mRNA levels after 24 h (Figure 2A and Figure S2). In the intestine, tnfα, *il6*, and *romo1* were positively regulated by rSpike in both periods (6 h and 24 h) compared to the healthy group (Figure 2B and Figure S3). In contrast, *coa1* mRNA levels increased significantly after 6 h and *il1b* after 24 h of rSpike inoculation (Figure 2B and Figure S3).

Regarding the liver, the PBM treatment restored *coa1* and *romo1* gene expression after 6 h compared with subjects injected with rSpike (Figure 2C and Figure S4). PBM intervention also modulated the expression of *il6*, *nfkbiab*, and *slc2a1a* genes after 24 h in the liver tissue (Figure 2C and Figure S4). However, no statistically significant difference was observed between the groups evaluated in *slc2a1a* and *tnfa* mRNA levels after 6 h and 24 h, respectively (Figure S4).

PBM treatment also significantly decreased the mRNA levels of *romo1* and *nfkbiab* genes relative to rSpike in both periods evaluated for testis tissue (Figure 2D and Figure S5), whereas *il1b* and *coa1* were negatively modulated only after 24 h (Figure 2D and Figure S5). There was no significant statistical difference in *tnfa* and *slc2a1a* expression levels after laser intervention according to Figure S5. Finally, the genes *romo1* and *tnfa* were negatively modulated by photobiomodulation in both periods for muscle tissue (Figure 2E and Figure S6). At the same time, *nfkbiab* mRNA levels were responsive only after 6 h (Figure 2E and Figure S6). According to statistical analysis (Figure S6), no statistical difference was observed in the expression of *il1b*, *il6*, *coa1*, and *slc2a1a* in laser-treated subjects compared to individuals treated with rSpike + PBM (Figure 2E and Figure S6).

The data in a heat map and genes were hierarchically clustered using Pearson’s correlation and the distance metric for all tissues (Figure 2). The results reveal the relationship between the treatments with distinct clusters for each gene expression, with a positive correlation between them.

### 2.3. The Metabolic Signature in a Zebrafish Model of CSS-Associated with COVID-19

**Figure 3:**
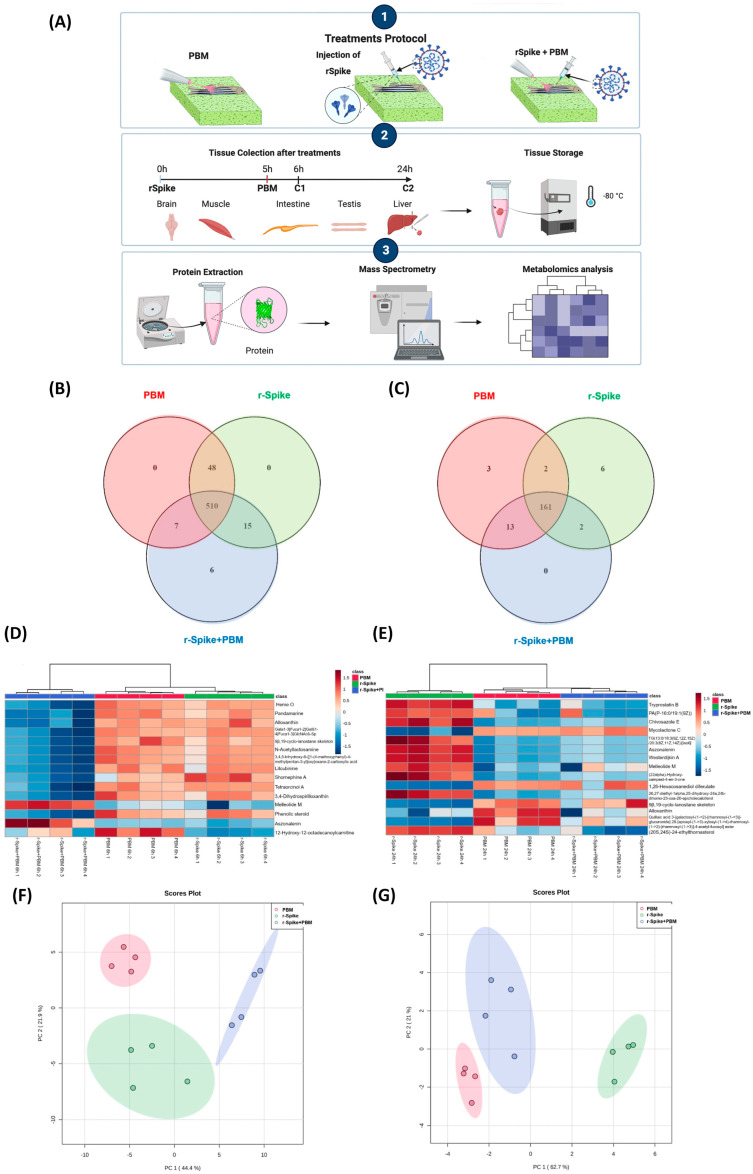
Metabolomics experimental protocol and unsupervised data analysis. (**A**) Venn diagram of the features distributed among each group of the 6 h treatment; (**B**) Venn diagram of the features distributed among each group of the 24 h treatment; (**C**) Heatmap of the top-15 most relevant features according to ANOVA of the 6 h samples; (**D**) Heatmap of the top-15 most important features according to the VIP score of the 24 h samples; (**E**) PCA of the 6 h groups; (**F**) PCA of the 24 h groups; (**G**) impacted metabolic pathways.

A metabolomics analysis evaluated the PBM treatment in zebrafish exposed to the rSpike protein. Therein, 24 samples were distributed into three different groups, (i) the control (PBM), (ii) rSpike, and (iii) (rSpike + laser), and two different time points, 6 and 24 h, were analyzed (Figure 3A). For the 6 h treatment group, 586 features were initially detected on the samples, from which 510 features were present in all sample groups, and 6 features were only present in the (rSpike + PBM) group (Figure 3B). On the other hand, 187 features were detected in the 24 h samples, 161 features were present in all groups, and 6 and 3 features were only present in the rSpike and PBM groups, respectively (Figure 3C). Figure 3D,E presents the Hierarchical Clustering Heatmap analysis from the 6 and 24 h groups. The Heatmaps show the patterns of the top-15 features ranked by relevance for the separation (based on ANOVA analysis) within the control (PBM), rSpike, and (rSpike + PBM) groups.

### 2.4. Metabolic Changes Induced by PBM Treatment in Zebrafish

Although the Heatmaps (Figure 3D,E) presented a clear separation between the groups, Principal Component Analysis (PCA) was also carried out on the samples for features filtering (variable reduction), samples comparison, and for group distribution verification along the principal components’ axis. The PCA analysis for both time points after PBM (Figure 3F,G) clearly shows the groups completely separated, corroborating with the Heatmaps analysis, and indicating a different metabolic panel in each studied group.

**Figure 4:**
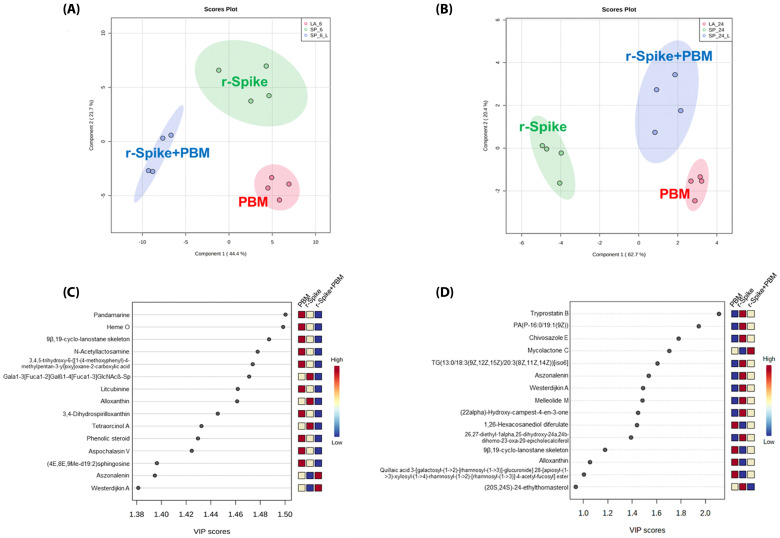
Supervised data analysis from metabolomics data to differentiate the PBM, rSpike, and (rSpike + laser) groups. (**A**) PLS-DA of the 6 h groups; (**B**) PLS-DA of the 24 h groups; (**C**) Variable importance for the projection (VIP) graph for the 6 h groups’ comparison; (**D**) VIP graph for the 24 h groups’ comparison.

Notwithstanding, for metabolites ranking, Partial Least Squares Discriminant Analysis (PLS-DA), a supervised clustering analysis was applied to the data (Figure 4A,B) to obtain the list of the most critical metabolites (Figure 4C,D). PLS-DA models were cross-validated to verify their applicability and avoid model overfitting. Therefore, permutation tests were performed on the models, resulting in predicted *p*-values of 0.012 and 0.013 for the 6 and 24 h timepoints, respectively. The models’ cross-validation was also performed. It resulted in a predictivity coefficient (Q^2^) higher than 0.80 in both time points, implying a good prediction capability for the PLS-DA models.

**Figure 5:**
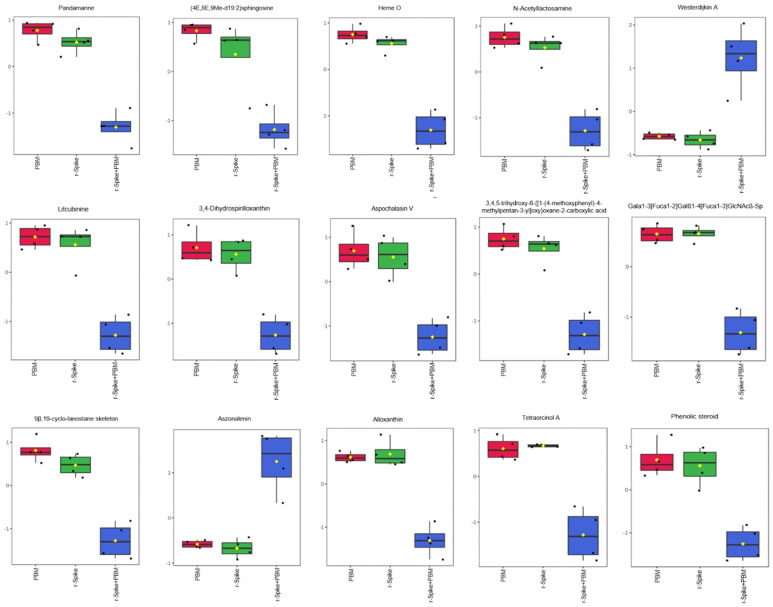
Boxplot plots of the most critical metabolites to differentiate the PBM, rSpike, and (rSpike + laser) after 6 h of treatment. Boxplot graphs referring to the 15 primary metabolites differentially expressed between groups after 6 h of treatment.

**Figure 6:**
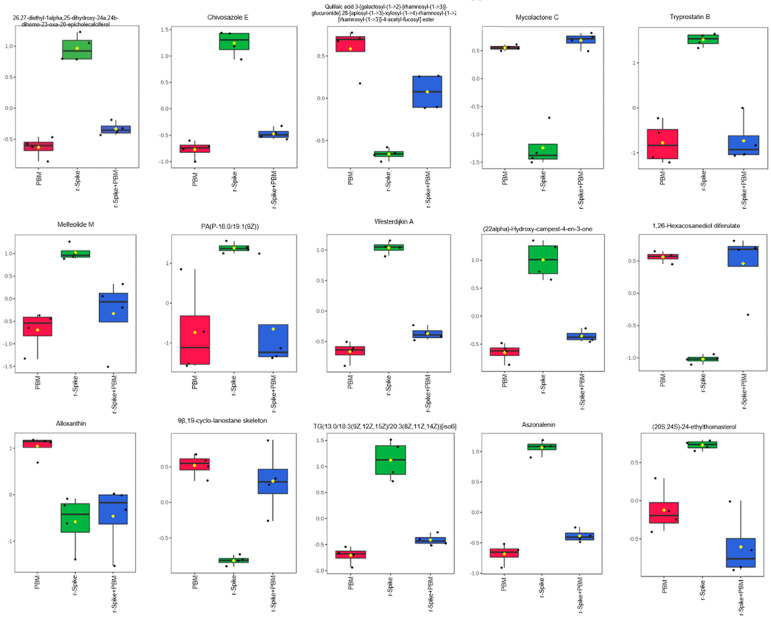
Boxplot plots of the most critical metabolites to differentiate the PBM, rSpike, and (rSpike + laser) after 24 h of treatment. Boxplot plots refer to the 15 primary metabolites expression between groups after 24 h of treatment.

For better visualization of the top-15 metabolites distribution within the groups, boxplot analysis was carried out for the 15 most essential metabolites for both time points (Figure 5 and Figure 6). Pandamarine and Heme O can be highlighted among the essential metabolites in the 6 h time point groups. In comparison, Tryprostatin B and PA (P-16:0/19:1(9Z)) can be highlighted as most important for disentangling the 24 h timepoint groups.

The boxplots of the first timepoint (6 h, Figure 5) indicate that the rSpike protein insertion in the animals did not affect the animal’s metabolism in just a few hours, as shown by the similar behavior between the control PBM alone and rSpike alone groups. Notwithstanding, the combination of rSpike + laser showed a different behavior on the metabolite levels. For the 6 h timepoint, pandamarine levels; heme O; 9β, 19-cyclo-lanostane; N-acetyllactosamine; sparfloxacin; litcubinine; 3,4-dihydrospirilloxanthin; ganglioside GM1 (d18:0/24:1(15Z); SSEA-3 antigen (d18:1/18:0), and (4E, 8E, 9Me-d19:2) sphingosine were higher in the control (PBM) and rSpike samples, while eplerenone and westerdijkin A were higher in the (rSpike + laser) samples, but their values were decreased in the control (PBM) and rSpike groups (Figure 4A). On the other hand, the 24 h timepoint boxplots (Figure 6) clearly shows a significant difference between PBM and the rSpike-treated groups.

**Figure 7:**
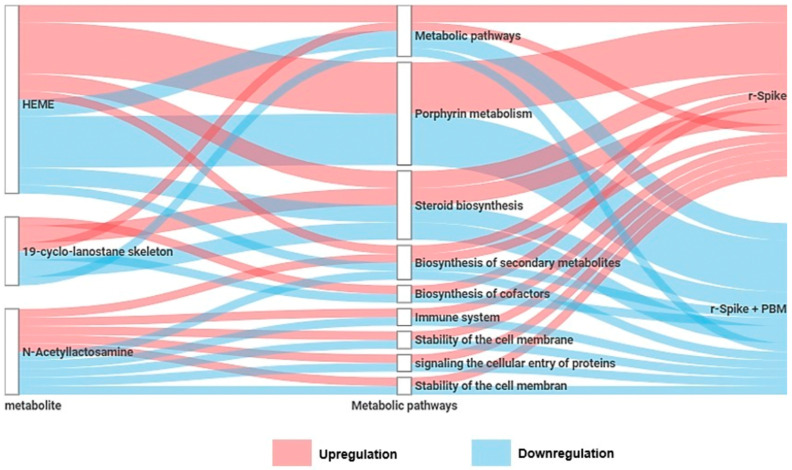
Summary of the metabolites and metabolic pathways differentially expressed between the recombinant Spike protein (rSpike) and PBM treatment.

After 24 h (Figure 6), the tryprostatin B; melleolide M; PA(P-16:0/19:1(9Z)); westerdijkin A; chivosazole E; epivitamin D3 (26,27-diethyl-1alpha,25-dihydroxy-24a,24b-dihomo-23-oxa-20-epivitamin D3/26,27-diethyl -1alpha, 25-dihydroxy-24a, 24b-dihomo-23-oxa-20-epicholecalciferol); (22alpha)-Hydroxy-campest-4-en-3-one; aszonalenin; (20S,24S)-24-ethylthornasterol; and TG (13:0/18:3(9Z,12Z,15Z)/20:3(8Z,11Z,14Z) levels were higher in rSpike groups, compared to the control PBM and rSpike + laser groups. Mycolactone C; 1,26-hexacosanediol diferulate; and quillaic acid levels were decreased in the rSpike group compared to the control PBM and (rSpike + laser) groups. Quillaic acid and alloxanthin were the only metabolites with a considerable difference between PBM and (rSpike + laser) groups. As a result, the most impacted metabolic pathways were related to steroid metabolism, the immune system, and lipid metabolism (Figure 7).

### 2.5. PBM Cell Repairing Effect for CSS-Associated Treatment in the Zebrafish Model

**Figure 8:**
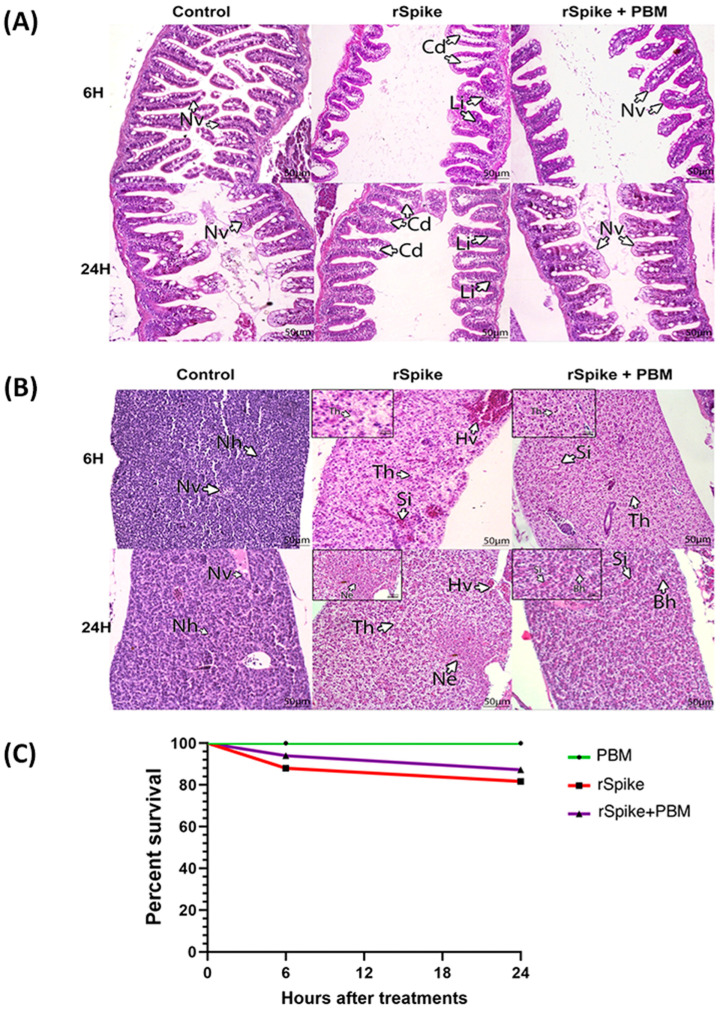
Characterization of acute tissue inflammation induced by rSpike in zebrafish male adults. Histopathological analysis of intestine (**A**) and liver (**B**) of zebrafish injected with recombinant Spike protein (rSpike) and PBM. The evaluations occurred after 6 and 24 h. All sections were stained with Hematoxylin and Eosin. Scale: 40 μm. Arrows indicate Nv: Normal villi; Cd: dilated vasa chylifer; Li: lymphocytes, Nh: normal hepatocytes; Hv: hyperemia of vessels; Th: turgid hepatocytes; Si: sinusoids; Ne: necrosis; Bh: basophil hepatocytes. (**C**) Cumulative Kaplan–Meier probability curve indicating the survival rate of zebrafish males submitted to different treatments: PBM (control), injected with recombinant Spike protein (rSpike), and (rSpike + PBM).

Several morphological alterations compatible with an inflammatory process were observed (Figure 8A,B). More precisely, after 6 h of evaluation, liver tissue obtained from fish treated with rSpike showed inflammatory features with the presence of dilated blood vessels, typical of hyperemia, as well as basophilic and turgid hepatocytes, characteristic of the situation of hydropic degeneration and cell edema (Figure 8B).

Furthermore, some fish analyzed after 24 h of inoculation presented hepatocellular necrosis as well as the same inflammatory characteristics (Figure 8B). The histopathological analysis of the intestinal tissue showed characteristic changes of lymphangiectasia, with dilatation of the vasa chylifera, as well as the occurrence of lymphocytic cells present in the intestinal villi (Figure 6A). Interestingly, after 24 h, PBM treatment restored the inflammatory process previously observed in subjects injected with rSpike in the intestinal tissue (Figure 7A). This response profile was not observed in the other tissues evaluated.

In the sequence, we compared the survival rate of individuals treated with PBM after rSpike inoculation with those who received rSpike injection (Figure 8C). Individuals injected with rSpike showed a decrease in survival rate after the first 6 h of inoculation from 88% to 81.7% after 24 h of inoculation (Figure 8C). Moreover, the lethality was significantly increased when compared to the control subjects, with a survival rate of 100% in both periods evaluated. It was observed that treatment with PBM reduced the mortality rate of individuals injected with rSpike after the first 6 h of application. The survival rate increased from 87.28% to 94% after 24 h. (Figure 8C).

## 3. Discussion

The process of virus infection and replication in host cells induces cellular oxidative stress, which is responsible for promoting the secretion of important pro-inflammatory factors, or “cytokine storms” [17,18,19]. Therefore, alternative treatments are needed to reduce the level of inflammation and pathology caused by COVID-19. In this sense, we aimed to uncover the gene expression and identify which metabolites are involved in the acute inflammatory response of COVID-19 and evaluated the action of red PBM as a potential treatment for inflammation generated by COVID-19 using zebrafish as a model.

Several studies have shown that the zebrafish is an excellent translational model for evaluating aspects of the “cytokine storm” [8,31,32]. Here, we show a series of sequential experiments on a zebrafish of COVID-19-associated cytokine storm syndrome using a recombinant spike protein from SARS-CoV-2. Our data demonstrates higher levels of pro-inflammatory markers, such as, *il-1β*, *il-6*, *tnf-*α, and *nfkbiab* in subjects injected with the rSpike protein compared to healthy subjects in most tissues. Studies on SARS-CoV-2-mediated cytokine induction also report positive regulation of these markers [17,18,19], and the association of the *nfκbiab* marker to activate the “cytokine storm” [33,34,35]. Additionally, we decided study these markers due to their involvement with the inflammasome process, which is considered to be the central role of inflammation in COVID-19 cases [36,37,38,39].

Moreover, the secretion of *IL1b*, *IL6*, and mediators, such as tumor necrosis factor (TNF-α), are also associated with the stimulation of hyper tissue inflammation and rapid disease progression in high-risk patients [15,16,17,18,19]. Among the other contributors to inflammasome activation, oxidative stress has independently been implicated in COVID-19 [40,41,42]. It is also accepted that the activation of the cellular immune system, as well as increased glucose uptake (hyper glucose) in COVID-19 patients, worsen the inflammatory response by increasing intracellular ROS production and stimulating the secretion of pro-inflammatory cytokines [43,44,45,46,47].

Upon SARS-CoV-2 binding to ACE2, multiple modifications occur to host homeostasis, including the promotion of the inflammatory response and deregulation in the renin-angiotensin system, resulting in the generation of ROS and a rise in oxidative stress. Moreover, ROS can interfere through NFKB and activate NLRP3 and increase the expression of IL-1 and IL-6 [48,49]. Increased ROS in the mitochondria and its consequent cellular toxicity is also related to COX1 enzyme dysfunction [50,51], a key enzyme in the catalytic production of inflammatory mediators such as prostaglandins (PTGES) [52]. Moreover, *cox-1*, *cox-2*, and PTGES have been shown to be upregulated in peripheral blood mononuclear cells isolated from patients with COVID-19 compared with healthy controls [53]. Nonetheless, other studies showed the involvement of *slc2a1a* (gene synonyms *glut1*) in several inflammatory and septic conditions. As happens in a patient with severe COVID-19, there is reprogramming of glucose metabolism, associated with increased expression of the ubiquitous sodium-independent glucose transporter 1 (*glut1*) in immune and non-immune cells, increased expression of glycolytic enzymes which contributes to the increase in glucose uptake [47]. In agreement with the pertinent literature, our study also confirmed that rSpike positively modulated mRNA levels of *slc2a1a* (cell membrane glucose transporter), as well as *ros* and *coa1* in various tissues (Figure 2).

Similarly, the protein–protein interaction prediction among SARS-CoV-2 and *D. rerio* proteins shows the biological pathways that can modify the normal function in an infection with SARS-CoV-2 and the alteration of the expression of related genes (Figure 1B). Among them, we can highlight the involvement of *slc2a1a*, *slc2a1b* (cell membrane glucose transport), interleukins- *il1b*, and *il6* (pro-inflammatory pathway), *nfkbiab*, and *coa1* (mitochondrial respiratory chain) in COVID-19. Collectively, our data support the hypothesis that the pathogenesis of rSpike is also related to the glycolytic pathway and oxidative stress in zebrafish. In this regard, rSpike stimulates the “cytokine storm” and substantially promotes the inflammatory process that plays a role in the development of the disease. Taken together, elevated inflammasome activation, aberrant oxidative stress response, and reprogramming of glucose metabolism were all strongly correlated with rSpike inflammation disease outcome, supporting our focus in highlighting the involvement of these markers.

In humans, several studies have reported the importance of PBM as an anti-inflammatory treatment and stabilizing immune responses in various diseases [54]. Considering the anti-inflammatory and biostimulatory effects of PBM, it seems to be a reasonable approach to be applied to controlling COVID-19 symptoms. Our findings revealed that PBM could be helpful in reducing the intestine inflammation and promoting the regeneration of damaged tissue after the inoculation of rSpike and increasing the survival rate in zebrafish (Figure 7). The most widespread findings of the inoculation with rSpike were lesions in the circulatory and digestive systems while PBM could increase oxygenation indirectly in order to rehabilitate the affected organs.

Another interesting result is that PBM treatment downregulates the mRNA levels of cytokines with inflammatory capacity (*il1b* and *il6*), including cellular respiration (*coa1*) and cell membrane glucose transporter markers (*slc2a1a*) (Figure 2). Moreover, PBM significantly reduces *tnfa* and *nfkbiab* expression levels compared to the rSpike group, suggesting the therapeutic potential of intervening to suppress inflammatory pathologies triggered by *tnfa*. Thus, infra-red laser by PBM is recommended in this regard. In the literature, many in vitro and in vivo clinical studies also report the action of PBM on inflammatory processes [55,56,57]. Positive effects are mainly found in the negative regulation of pro-inflammatory cytokines (IL-1β, IL-6, IL-2), and markers of cellular respiration (*cox2*) at both 660 nm and 684 nm wavelengths [28,30,58,59,60,61,62].

Aimbire et al. [60], also denotes that PBM significantly reduces TNFα and Nfkβ levels compared to the control group, suggesting therapeutic potential in suppressing TNFα in various inflammatory pathologies in the clinical area. Similarly, recent systematic reviews also suggest that PBM at blue wavelengths (450, 454, and 470 nm) favor lung tissue regeneration in patients with asthma as well as pneumonia triggered by COVID-19 [63,64,65,66,67,68]. Patients treated with PBM showed rapid recovery of pulmonary status, without the need for ICU admission or mechanical ventilation after treatment [68].

Moreover, to the best of our knowledge, little is known about the metabolic mechanisms of rSpike and photobiomodulation. Metabolic changes induced by rSpike could be observed by metabolomics, particularly for the 24 h groups, in which the levels of the most critical metabolites differed from the control group (PBM) (Figure 4). However, it is interesting to note that for the 6 h groups, the levels of several metabolites were similar for the PBM and rSpike groups, indicating that the metabolic effect of rSpike inoculation is not immediate. Moreover, the treatment with PBM on the rSpike-inoculated animals (rSpike + PBM group) decreased most metabolites levels similar to those recorded in the control group (PBM). This effect suggests that treating PBM in animals with COVID-19-associated cytokine storm syndrome can restore inflammatory mediators’ metabolism and gene expression to the basal condition, virtually eliminating the systemic rSpike metabolic effects. Furthermore, this effect suggests that PBM therapy might protect against SARS-CoV-2’s inflammatory response.

Apart from the specific metabolic differences, the most exciting discussion emerges from the affected metabolic pathways in the different analyzed groups. We discovered a modulation of lipid metabolism, especially glycerophospholipid metabolism, modulation of porphyrin metabolism, biosynthesis of cofactors, steroid biosynthesis, and immune system metabolism (Figure 4; Figure 7). Studies have found that viruses, such as SARS-Cov2, can enhance lipid biosynthesis [69,70,71,72,73]. Viruses, including coronaviruses, require lipid droplets (LDs) during their replication phase. LDs store mainly triacylglycerols utilized for membrane formation but also supply the cells with essential lipids for metabolic energy, signaling molecules, and inflammatory mediators, resulting in cytokine production [74,75,76,77,78]. Due to their importance for viral replication, LDs have been proposed as therapeutic targets against SARS-CoV-2 infection since interfering with lipid synthesis and storage would decrease the infection. However, it was shown that LDs support SARS-CoV-2 replication and increase inflammatory mediators during the infection [69,79,80]. Furthermore, it was found that the inhibition of triacylglycerol synthesis decreased viral replication through the expression of pro-inflammatory mediators, such as leukotriene B2, TNF-α, and IL-6, leading to cell death [81].

Another altered pathway was steroid biosynthesis, closely related to the human stress response. Upregulation in such a pathway triggers the hypothalamic-pituitary-adrenal axis (HPA), inducing the secretion of a series of corticosteroids, which have anti-inflammatory properties, helping the immune system to fight the viral infection [82]. However, a high level of steroids can be harmful to the patients, inducing a series of discomforts and affecting the mental health of patients. PBM treatment could downregulate the steroid metabolism (Figure 4), restoring it to control levels. The porphyrin metabolism was upregulated in the rSpike group compared to the control (PBM) and treatment (rSpike + PBM) groups. Previous studies have also reported alterations in the porphyrin metabolism, suggesting that the SARS-CoV-2 infection can change the hemoglobin synthesis, reducing the respiratory capacity of the patient. Notwithstanding, the proposed treatment with PBM was able to reverse this behavior in zebrafish models.

**Figure 9:**
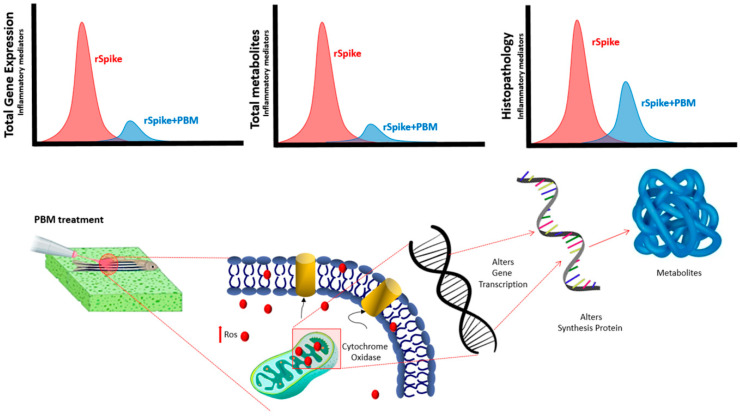
Summary of the effects of recombinant Spike protein (rSpike) and PBM treatment in gene expression, metabolites and histopathology. Analyses were performed using bioassays at 6 and 24 h after treatments.

In conclusion, here, we tried to determine the therapeutic potential of PBM for inflammatory state modulation in a zebrafish model of COVID-19-associated cytokine storm syndrome (Figure 9). The results showed that rSpike was responsible for generating systemic inflammatory processes with significantly increased levels of pro-inflammatory and oxidative stress mRNA markers, with a pattern similar to those observed in COVID-19 cases in humans. On the other hand, PBM treatment was able to decrease the mRNA levels of pro-inflammatory and oxidative stress markers compared with rSpike. Conversely, PBM reduced the intestine inflammation stimulated previously by rSpike injection. These results are in concordance with a significant increase in the survival rate of rSpike-inoculated individuals. In summary, this study suggests that PBM can be used as a novel therapeutic agent for patients with COVID-19 by regulating the inflammatory response and promoting cellular and tissue repair of injured tissues. Further in-depth studies are needed to fully address this issue.

## 4. Materials and Methods

### 4.1. Cloning, Expression, and Recombinant SARS-CoV-2 Spike Protein Purification

The cloning, expression, and purification of the N-terminal region of the Spike protein were previously described [8]. Methodology details are in a supplementary file (Figure S7). The SARS-CoV-2 Spike DNA fragment corresponding to the residues from 16 to 165 (rSpike) was amplified by PCR using a SARS-CoV-2 cDNA. The purified PCR product was digested using AnzaTM restriction enzymes NheI and BamHI (Thermo Fisher Scientific, Waltham, MA, USA), and the same pair of enzymes were used to digest the expression vector pET-28a. The digested and purified plasmid was used to ligate the rSpike DNA fragment. Digestion tests confirmed the positive clones. This cloning results in a fusion of seven histidine tags at the N-terminal portion of the protein. rSpike was purified by expression of the protein in Escherichia coli strain BL21(DE3) or BL21 StarTM (DE3), followed by two steps of purification using HisTrap Chelating HP column (GE Healthcare Life Sciences, Chicago, IL, USA) and HiLoad 16/600 Superdex 75 pg (GE Healthcare Life Sciences) size exclusion chromatography. The purified protein (7 M urea, 50 mM MOPS, 200 mM NaCl, 1 mM EDTA, pH 7.0) was concentrated using Amicon Ultra-15 Centrifugal filters (Merck Millipore, Milwaukee, WI, USA) with a 3 kDa membrane cut-off.

### 4.2. Zebrafish Maintenance

Adult male zebrafish were bred and raised in the aquarium facility at the Department of Structural and Functional Biology, Institute of Biosciences, São Paulo State University (Botucatu, Brazil). Fish used for the experiments were obtained from natural crossings and raised according to standard methods. Fish were kept in 10 L tanks in a recirculation system (28 °C; pH 7.6; conductivity of 750 μS) under a 14 h:10 h (light, dark) photoperiod. Salinity, pH, dissolved oxygen, and ammonia were monitored daily.

### 4.3. Inoculation of SARS-COVID-2: rSpike

Adult zebrafish males (*n* = 80) were injected abdominally with 10 μL of recombinant Spike protein (rSpike). After inoculation, the fish were separated in 10 L aquariums at 28 °C, pH 7.6, under a 14 h:10 h (light, dark) photoperiod for subsequent treatment with a low-level laser. Inoculation of rSpike was performed from a solution containing 1 μg of rSpike diluted in 10 μL of a pH 7.5 buffer (urea 7 mol L^–1^, Tris-HCl 50 mmol L^−1^, NaCl 200 mmol L^−1^, and EDTA 1 mmol L^−1^). Before the treatment, all fish were anesthetized with 120 μg/mL tricaine methanesulfonate (Sigma-Aldrich, San Luis, MI, USA). A group of control animals received injections containing only the dilution buffer. The production of recombinant Spike protein (rSpike) was obtained previously [8].

### 4.4. Photobiomodulation (PBM) Therapy

To elucidate the role of PBM with low-level laser as a potential therapeutic in pathologies originating from SARS-CoV-2, adult males injected with rSpike (*n* = 40) were subjected to PBM after 5 h of inoculation. Adult males (*n* = 40) were subjected only to treatment with a low-power laser as a control group. For this purpose, the low-power laser device, Therapy XT (DMC, São Carlos, Brazil), with an output power of 100 mW, emitting at 660 nm and energy density of 2 J cm^–2^, was used. After treatment with PBM, the fish were kept in a 10 L tank at 28 °C, pH 7.6, and under a 14 h:10 h (light, dark) photoperiod.

After the 6 and 24 h periods, the experimental groups were euthanized by overdose with benzocaine hydrochloride (250 mg) previously dissolved in ethanol and then mixed with water. Collections of different tissues (the brain, testicle, intestine, liver, and muscle) were performed for each experimental group (1) control, (2) rSpike, (3) rSpike + laser for gene expression, as well as collections from adult subjects for histopathological analysis.

### 4.5. RNA Extraction and Gene Expression (qPCR)

The gene expression of factors involved in signaling and responding to inflammatory processes was examined in zebrafish treated with COVID-19 (rSpike) to assess the therapeutic effect of low-level laser. For this, different tissues (the brain, testicle, intestine, liver, and muscle) were collected from adult males (*n* = 15) in each experimental group: (1) PBM (control), (2) rSpike, and (3) Spike + laser. The tissues were frozen in liquid nitrogen and stored at –80 °C until RNA extraction. The total RNA extraction process from each organ was performed according to the manufacturer’s specifications for the Trizol method (Invitrogen, Carlsbad, CA, EUA). cDNA was synthesized using a Superscript II kit according to the manufacturer’s protocols (Bio-Rad, Hercules, CA, USA).

The qPCR reactions were conducted with 10 μL 2X SYBRGreen Universal Master Mix, 2 μL forward primer (9 mM), 2 μL reverse primer (9 mM), 1 μL DEPC water, and 5 μL cDNA. The Cts were determined using StepOne Plus (Applied Biosystems, Thermo fisher, Waltham, MA, USA), and reactions were subjected to 40 cycles (5 min at 95 °C, 40 cycles of denaturation at 95 °C, for 30 s at 60 °C, and 30 s at 72 °C). The relative mRNA levels of genes involved in signaling processes and inflammatory responses were evaluated in the different experiments (Table S3). Primers were made using Primer Express 3.0 software (Software for Windows) based on sequences deposited in the public International Database of the National Center for Biotechnology Information (NCBI) (http://www.ncbi.nlm.nih.gov/ accessed on 25 August 2022). The relative expression of the genes studied was determined by the ddCt method. The mRNA levels (Cts) were calculated and normalized using the reference gene β-actin.

### 4.6. Protein–Protein Interactions with SARS-CoV-2

*Danio rerio* predicted protein–protein interactions with SARS-Cov-2 related to the subcellular location: the cytoplasm, membrane, and nucleus were recovered according to the Fernandes and collaborators 2022 [8]. The genes of interest were added for each subcellular location (denominated the related genes (*slc2a1a*, *slc2a1b*, *romo1*, *nfkb1*, *il1b*, *il6*, and *cox1*)). The interacted proteins and the genes were submitted to functional enrichment to identify gene ontology (GO) using the G:Profiler software v1.1 [83] based on the database of the *D. rerio*; the sub-networks of proteins and pathways (KEGG and REACTOME) were obtained with the Cytoscape software v1.1 [84]. The sub-networks were extracted according to the first neighbor of the related genes (*slc2a1a*, *slc2a1b*, *il1b*, *il6*, *nfkb1*, and *cox1*).

### 4.7. Mapping of RNA-Seq Libraries and Differential Gene Expression Analysis

RNA-seq raw data was obtained from human brain (GEO accession: GSE182297), heart (GEO accession: GSE36761 and GSE150316), liver (GEO accession: GSE61474 and GSE182297), and colon (NCBI BioProject accession: PRJNA646224), infected with SARS-CoV-2 and control samples, available at public databases. Initially raw reads served as input for Trimmomatic 0.40 [85], which performed quality filtering removing Illumina adaptor sequences, low quality bases (phred score quality > 20), and short reads (PE -phred33 ILLUMINACLIP:truseq.fa:2:30:10 LEADING:3 TRAILING:3 SLIDINGWINDOW:3:20 MINLEN:36). Trimming was followed by read error correction by the SGA *k*-mer-based algorithm [86], version 0.9.9 with standard settings. Next, the reads were mapped against the Genome Reference Consortium Human Build 38 (GRCh38) and the transcript abundance was quantified using Salmon [87]. Finally, the count data were direct to differential analysis with DESeq2 (version 1.34.0) R 4.1.2 package [88,89] with standard settings.

### 4.8. Histopathological Analysis

Histopathological analyses were performed on adult zebrafish males (*n* = 30/10 per group) subjected to the different treatments (1) PBM (control), (2) rSpike, (3) rSpike + laser collected over two periods (after 6 and 24 h). For this purpose, individuals from different experimental groups were fixed with 4% paraformaldehyde. The material was then dehydrated in an increasing series of alcohol (70% for 4 h; 95% for 4 h), infiltrated, and embedded in paraffin (Leica Wetzlar, Hessen, Germany). Histological sections 5 μm thick were obtained and stained with Hematoxylin and Eosin for observation of general cellular structures. The material was documented using a microscope with a JVC camera and a computer with Leica QWIN software v3.1.

### 4.9. Metabolomic Analysis and Metabolite Extraction

The animals were separated into three distinct groups: (i) PBM group, containing eight animals submitted to laser treatment; (ii) rSpike group, containing eight animals inoculated with recombinant Spike protein from the SARS-CoV-2 virus; and (iii) rSpike + PBM group, containing eight animals inoculated with rSpike and subsequently treated with PBM. Additionally, two time points were evaluated for each group, 6 and 24 h after rSpike inoculation. After euthanasia, the animals were immediately stored in a –80°C freezer until sample preparation. The extraction of metabolites was performed by Bligh and Dyer procedure (1959) [90], modified by Wu et al. (2008) [91]. Frozen specimens were individually homogenized using a FastPrep-24 homogenizer (MP BIOMEDICALS^®^, Santa Ana, CA, USA), using lysis matrices D (1.4 mm ceramic spheres) (MP BIOMEDICALS^®^, Santa Ana, CA, USA). For the homogenization solvent, a ratio of 1:4 (g:mL) of sample mass to volume of ice-cold solution A (MeOH/H2O 1:0.8 (g:mL)) was used. After homogenization, the mixture was transferred to a new container, where chloroform was added, resulting in a final ratio of MeOH/CHCl_3_/H_2_O of 2:2:1.8 (g/g/mL). The organic phase was collected, dried, and stored at –80 °C until the day of analysis.

### 4.10. Analysis by LC-MS

The dried extracts were resuspended in ethyl acetate prior to the analysis. The LC-MS analysis was performed using ultra-high-performance liquid chromatography (UHPLC Thermo Accela^®^, Germany) and a C18 column (ACQUITY HSS T3; 150 × 2.1 mm × 2.8 µm) heated to 40 °C. The injection volume was 15 µL, and the run was carried out with a flow of 350 µL/min. The mass spectrometer employed consisted of an LTQ Orbitrap Velos^®^ (ThermoScientific, Dreieich, Germany) operating in positive and negative modes, covering the range of 200–2000 *m*/*z* [92]. Quality control analyses were submitted to MS/MS applying CID fragmentation with 40 eV and analyzed every 7 runs.

### 4.11. Metabolomics Data Analysis

Data pre-processing was carried out with Mzmine 2.0 to remove noise signals, isotopes, and duplicated signals and perform the alignment and filtering of the signal. Then, sequentially, the features were analyzed using the MetaboAnalyst 5.0 platform (http://www.metaboanalyst.ca, accessed on 3 October 2022). Finally, the annotations of the most-impacting compounds were performed through Metlin, HMDB, mzCloud, and CEU Mass Mediator databases.

### 4.12. Statistical Analysis

GraphPadPrism 9.0 software (GraphPad Software, Inc., San Diego, CA, USA, http://www.graphpad.com, accessed on 15 October 2022) was used for all statistical analyses. Initially, the data were checked for deviations from the normality of variance and homogeneity of variance. Data normality was assessed by the Shapiro–Wilk test and homoscedasticity by the Bartlette test. The results were expressed as mean ± SE (standard error). One-way ANOVA followed by Tukey’s multiple comparisons test were employed. Different letters indicate significant differences (*p* < 0.05) between different treatment conditions. Correlation analyses were performed using Pearson’s test. The significance level adopted for all analyses was alpha = 0.05.

## Data Availability

The raw data supporting the conclusions of this article will be made available by the authors, without undue reservation, to any qualified researcher. The data are available from the author, I.F.R., upon request.

## Supplementary Materials

The supporting information can be downloaded at: https://www.mdpi.com/article/10.3390/ijms24076104/s1.
