# Supplementary material for: Photobiomodulation Reduces the Cytokine Storm Syndrome Associated with COVID-19 in the Zebrafish Model
Source: Int J Mol Sci. 2023 Mar 24;24(7):6104. doi: 10.3390/ijms24076104 (PMC10094635; doi:10.3390/ijms24076104)
Supplement: Supplementary file 1 [file ijms-24-06104-s001.zip › Supplementary Material.pdf]

Photobiomodulation Reduces the Cytokine Storm Syndrome Associated with COVID-19 in the Zebrafish Model

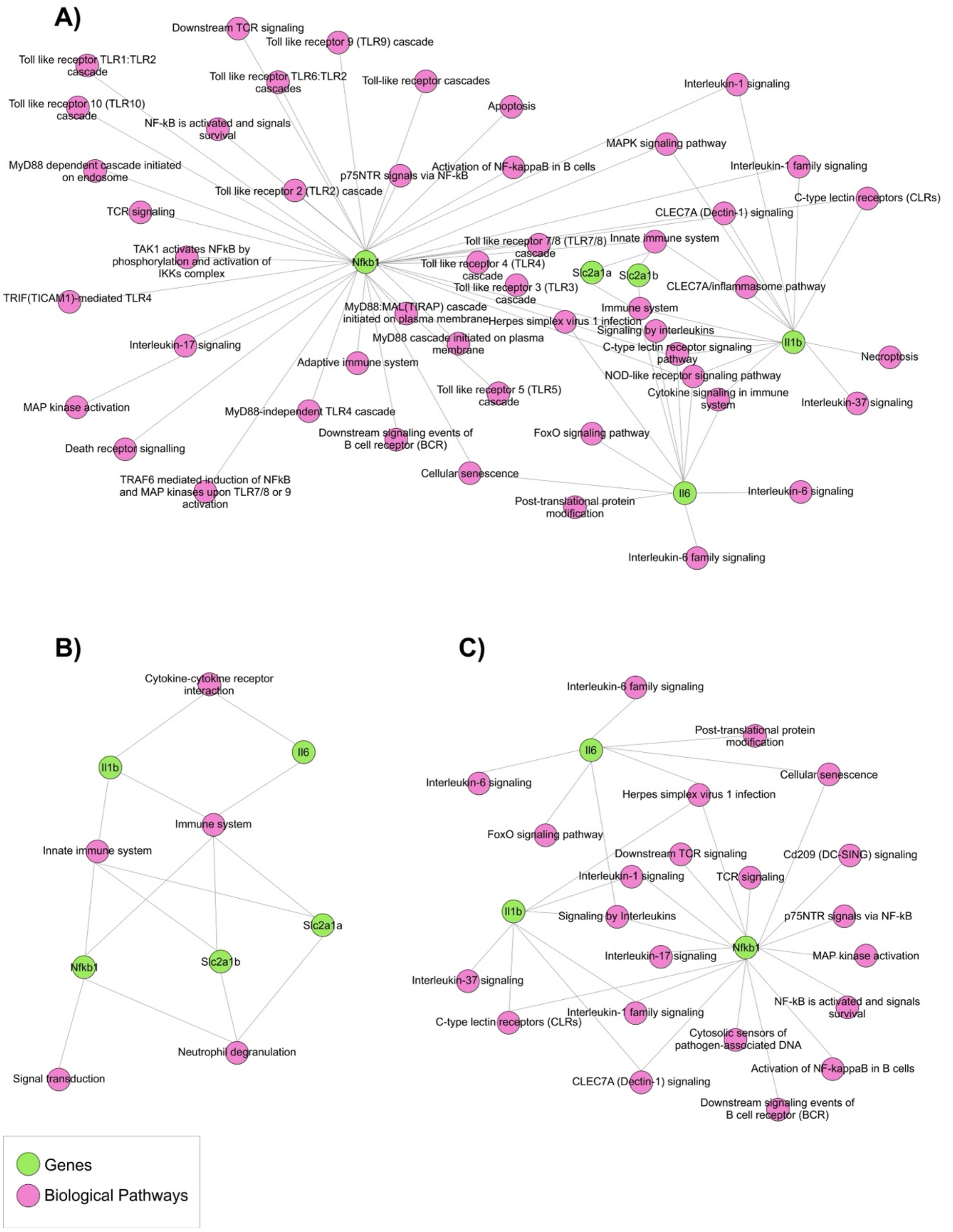

Supplementary Figure S1. Network of enrichment function of protein prediction interacted with SARS-CoV-2 and related genes. In **A)** proteins related to cytoplasm, in **B)** proteins related to membrane and in **C)** the proteins related to nucleus. The boxes are the legend: light green represents related genes and purple represent biological pathways. The networks were obtained with the Cytoscape software (Shannon et al. 2003).

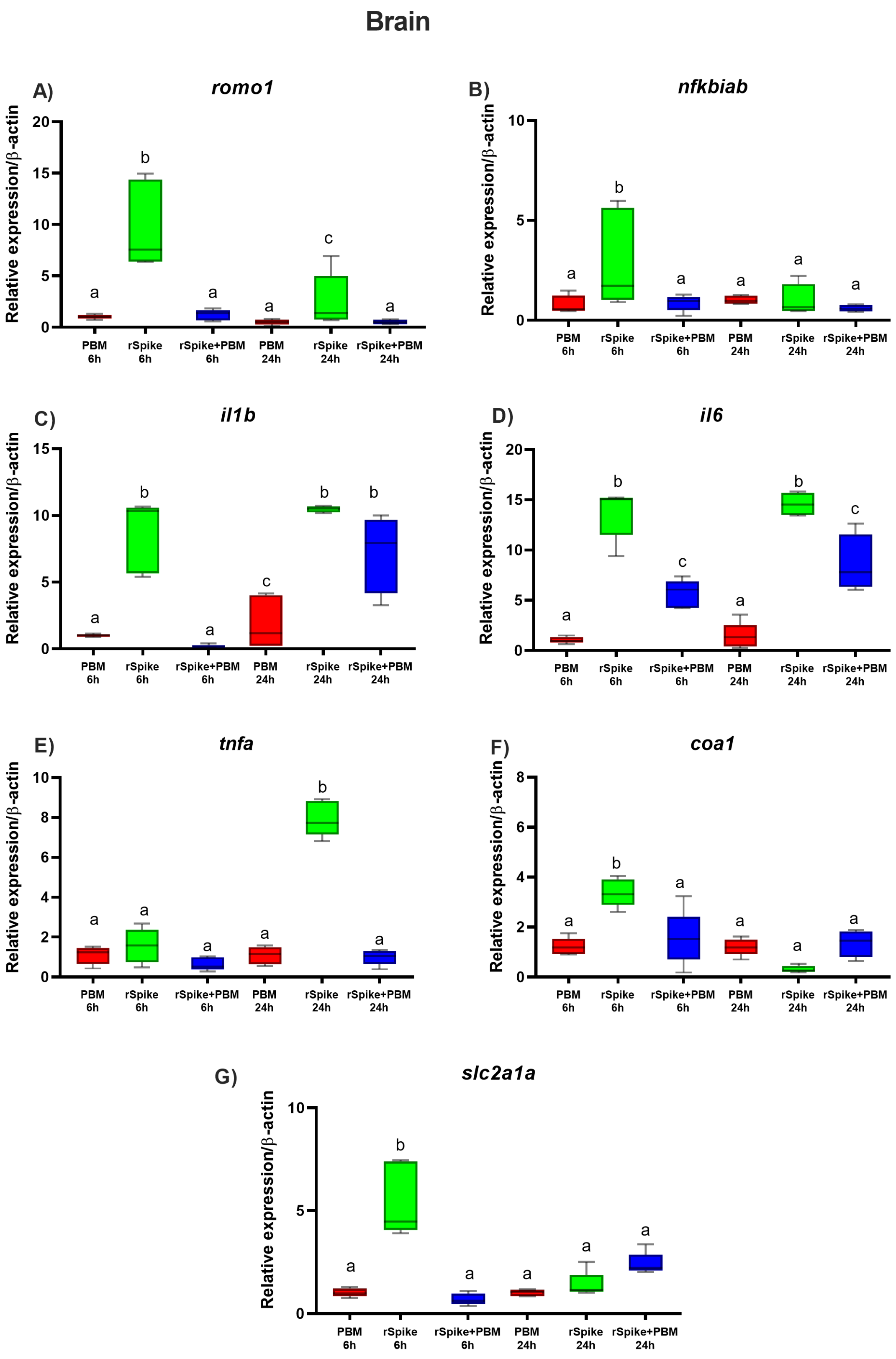

Supplementary Figure S2. Relative mRNA levels of **A**) *romo1*, **B**) *nfkbiab,* **C**) *il1b*, **D**) *il6*, **E**) *tnfa*, **F**) *coa1*, and **G**) *slc2a1a* in brain tissue of zebrafish males subjected to PBM (6h and L4h); injected with recombinant Spike protein (rSpike) and subsequently subjected to PBM (6h and 24h). Quantification was performed using the 2-ΔΔCt method and values were normalized by the respective β-actin values. One-way ANOVA followed by Tukey's multiple comparisons test; different letters indicate significant differences ($p < 0.05$) between different treatment conditions.

Intestine

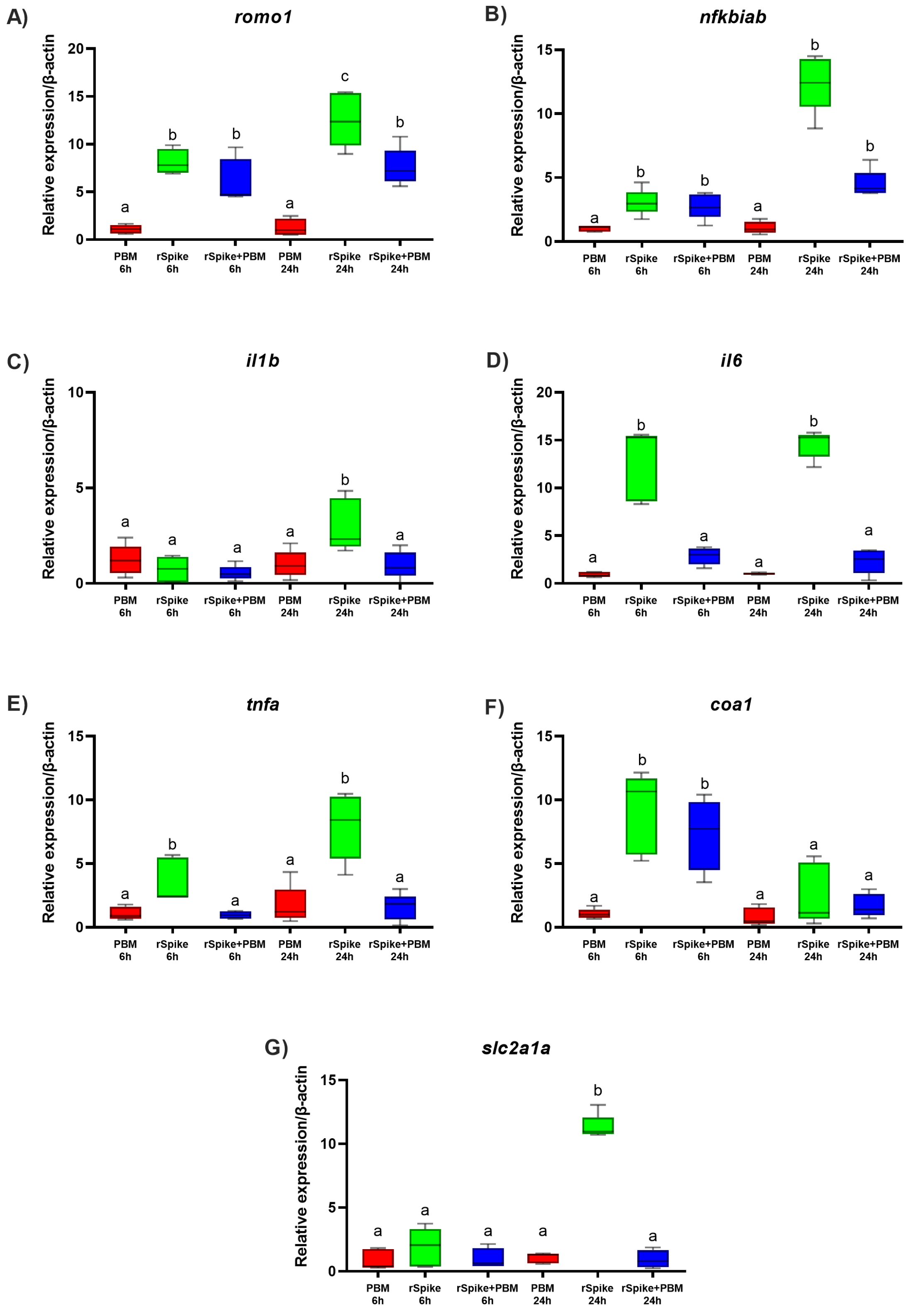

Supplementary Figure S3. Relative mRNA levels of **A**) *romo1*, **B**) *nfkbiab,* **C**) *il1b*, **D**) *il6*, **E**) *tnfa*, **F**) *coa1*, and **G**) *slc2a1a* in intestine tissue of zebrafish males subjected to PBM (6h and L4h); injected with recombinant Spike protein (rSpike) and subsequently subjected to PBM (6h and 24h). Quantification was performed using the 2-ΔΔCt method and values were normalized by the respective β-actin values. One-way ANOVA followed by Tukey's multiple comparisons test; different letters indicate significant differences ($p < 0.05$) between different treatment conditions.

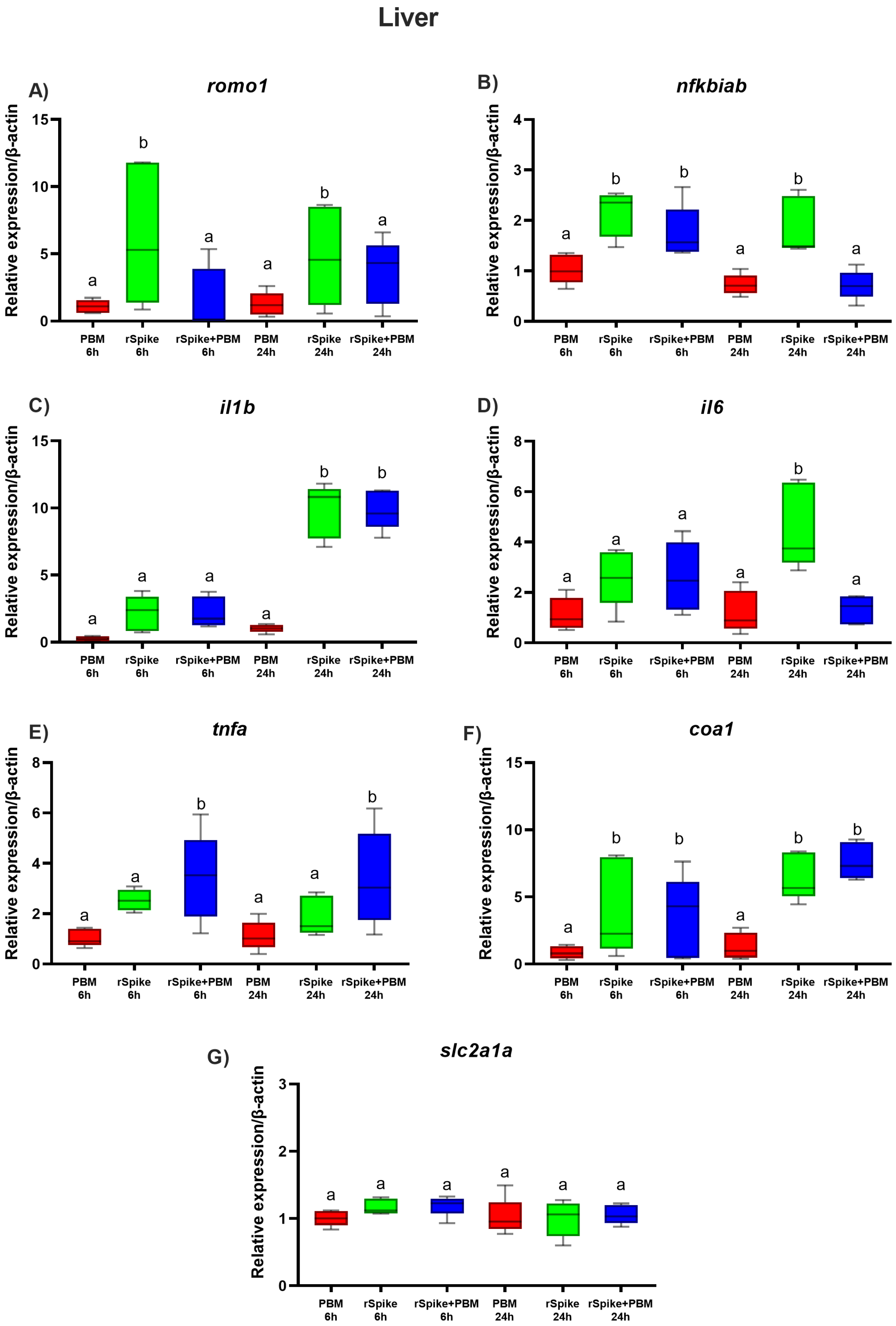

Supplementary Figure S4. Relative mRNA levels of **A**) *romo1*, **B**) *nfkbiab,* **C**) *il1b*, **D**) *il6*, **E**) *tnfa*, **F**) *coa1*, and **G**) *slc2a1a* in liver tissue of zebrafish males subjected to PBM (6h and L4h); injected with recombinant Spike protein (rSpike) and subsequently subjected to PBM (6h and 24h). Quantification was performed using the 2-ΔΔCt method and values were normalized by the respective β-actin values. One-way ANOVA followed by Tukey's multiple comparisons test; different letters indicate significant differences ($p < 0.05$) between different treatment conditions.

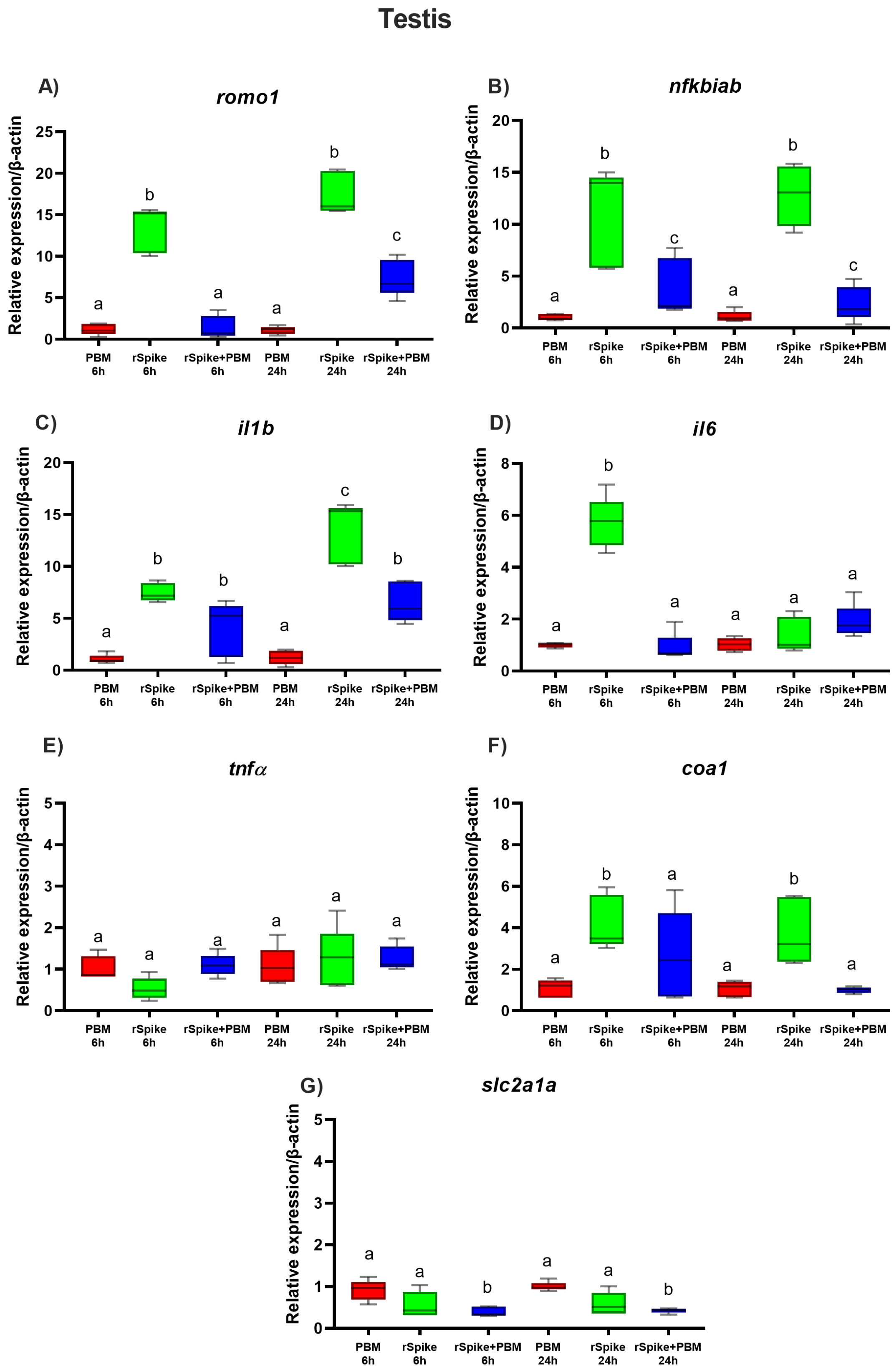

Supplementary Figure S5. Relative mRNA levels of **A**) *romo1*, **B**) *nfkbiab,* **C**) *il1b*, **D**) *il6*, **E**) *tnfa*, **F**) *coa1*, and **G**) *slc2a1a* in testis tissue of zebrafish males subjected to PBM (6h and L4h); injected with recombinant Spike protein (rSpike) and subsequently subjected to PBM (6h and 24h). Quantification was performed using the 2-ΔΔCt method and values were normalized by the respective β-actin values. One-way ANOVA followed by Tukey's multiple comparisons test; different letters indicate significant differences ($p < 0.05$) between different treatment conditions.

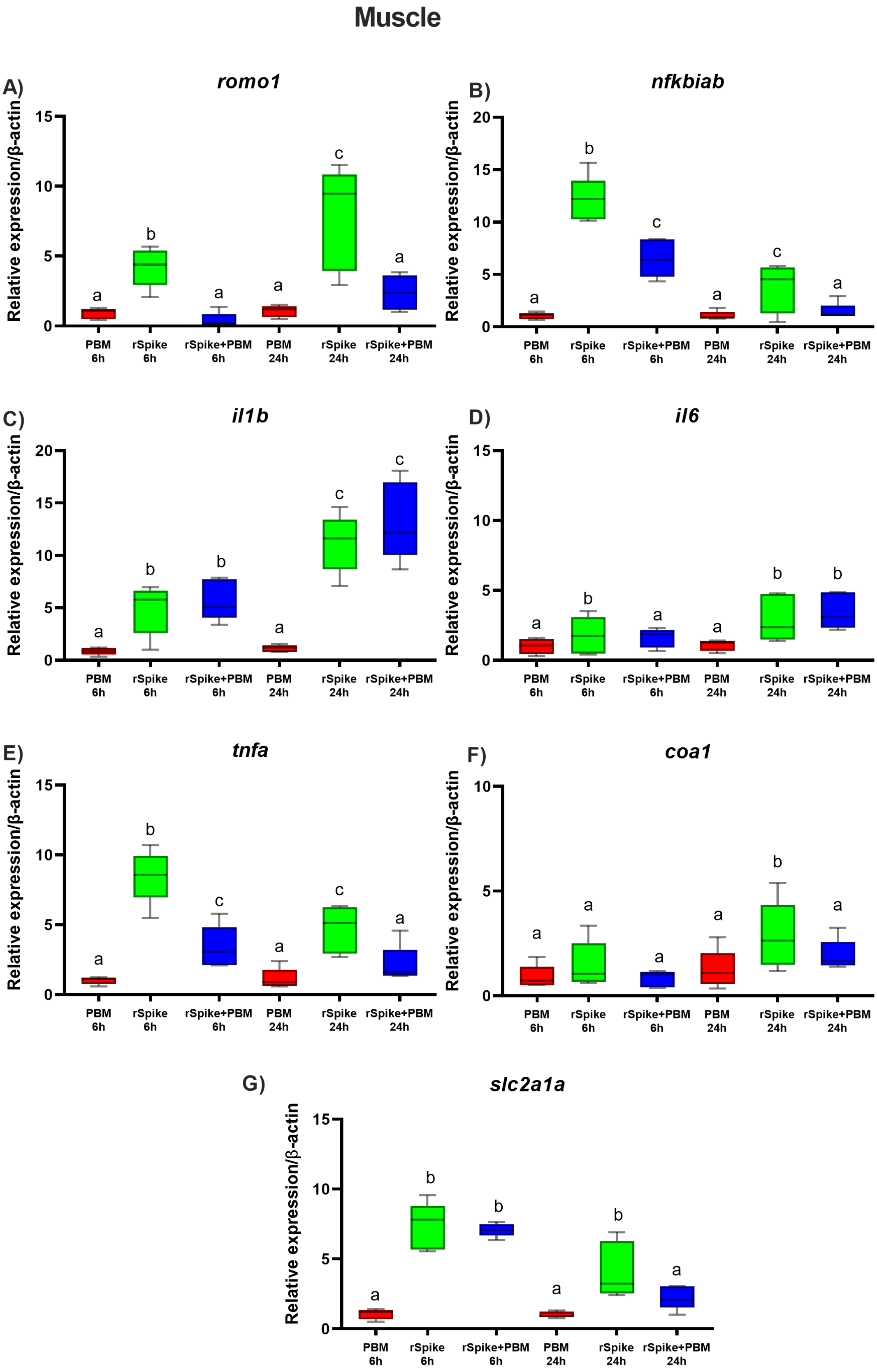

Supplementary Figure S6. Relative mRNA levels of **A**) *romo1*, **B**) *nfkbiab,* **C**) *il1b*, **D**) *il6*, **E**) *tnfa*, **F**) *coa1*, and **G**) *slc2a1a* in muscle tissue of zebrafish males subjected to PBM (6h and L4h); injected with recombinant Spike protein (rSpike) and subsequently subjected to PBM (6h and 24h). Quantification was performed using the 2-ΔΔCt method and values were normalized by the respective β-actin values. One-way ANOVA followed by Tukey's multiple comparisons test; different letters indicate significant differences ($p < 0.05$) between different treatment conditions.

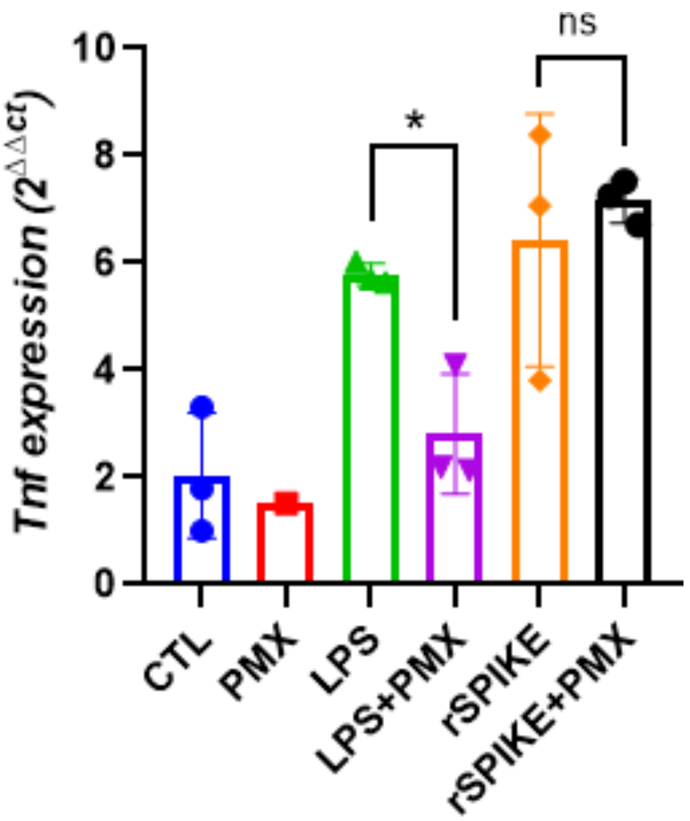

Supplementary Figure S7. Photobiomodulation reduces the cytokine storm syndrome associated with Covid-19 in the zebrafish model.

We confirm that our protein (rSpike) that there is no contamination by LPS. For this, J774A.1 cell line were used to analyze the neutralization of LPS itself and possible LPS contamination of recombinant Spike protein (rSpike) produced in E. coli. J774A.1 cells were cultured in DMEM high glucose containing 10%FBS, 100U/mL penicillin, 100ug/mL streptomycin, and 30mM HEPES. Cells were plated at a density of 5x105cells/well in the presence or absence of Polymyxin (PMX) B (10ug/ml, Sigma-Aldrich) and were stimulated with LPS (100ng/mL, Sigma-Aldrich) or rSpike (2.5ug/mL). Unstimulated cells in the presence or absence of PMX were cultured as a control. Cells were incubated at 37°C, 5% CO_2, for 8 hours. Then, the cells were collected and submitted to qPCRanalysis. The asterisk symbol (*) indicates statistically significant differences between transcript abundance ($p < 0.05$). ns: not significant.

Supplementary Table S1: Gene ontology enrichment analysis of PPI proteins and related genes data (in xlsx version).

Supplementary Table S2: Number of Gene Ontology retrieved from functional enrichment.

Subcellular localization	N° GO – functional enrichment	N° GO – related
**Cytoplasm**	116	48
**Membrane**	128	5
**Nucleus**	143	22

N° GO – related: functional enrichment terms that present one of the genes of interest.

Supplementary Table S3: Oligonucleotide sequences used for gene expression studies (qPCR).

Targets	Primers Forward (3'-5')	Primers Reverse (5'-3')	References
β-actin	AGACATCAGGGAGTGATGGT	CAATACCGTGCTCAATGGGG	[8]
coa1	CTGCCCAGTGGGTGAAATA	TGATGCTCCTTCAGTTGGTTC	Present study
slc2a1a	ATTTGAGAAAGCGGGGGTGT	TCGCTCCACTATGAACAGAGAA	Present study
il6	TCAACTTCTCCAGCGTGATG	TCTTTCCCTCTTTTCCTCCTG	Present study
il1b	TTCCCCAAGTGCTGCTTATT	AAGTTAAAACCGCTGTGGTCA	Present study
nfkbiab	CTCACCGAGGACGGAGACA	CTCTTCGGGATAACGCAATCA	Present study
tnfa	GCTGGATCTTCAAAGTCGGGT GTA	TGTGAGTCTCAGCACACTTCCATC	Present study
romo1	CGTCCGTGTGAAACAGTCCT	ACACTCACCGGCATCTTGTT	Present study
